# A *lex naturalis* delineates components of a human-specific, adrenal androgen-dependent, p53-mediated ‘kill switch’ tumor suppression mechanism

**DOI:** 10.1530/ERC-19-0382

**Published:** 2019-12-09

**Authors:** Jonathan Wesley Nyce

**Affiliations:** 1 ACGT Biotechnology, Collegeville, Pennsylvania, USA

**Keywords:** p53, TP53, DHEA, cancer prevention, vitamin C

## Abstract

We have recently described in this journal our detection of an anthropoid primate-specific, adrenal androgen-dependent, p53-mediated, ‘kill switch’ tumor suppression mechanism that reached its fullest expression only in humans, as a result of human-specific exposure to polycyclic aromatic hydrocarbons caused by the harnessing of fire – but which has components reaching all the way back to the origin of the primate lineage. We proposed that species-specific mechanisms of tumor suppression are a generalized requirement for vertebrate species to increase in body size or lifespan beyond those of species basal to their lineage or to exploit environmental niches which increase exposure to carcinogenic substances. Using empirical dynamic modeling, we have also reported our detection of a relationship between body size, lifespan, and species-specific mechanism of tumor suppression (and here add carcinogen exposure), such that a change in any one of these variables requires an equilibrating change in one or more of the others in order to maintain lifetime cancer risk at a value of about 4%, as observed in virtually all larger, longer-lived species under natural conditions. Here we show how this relationship, which we refer to as the *lex naturalis* of vertebrate speciation, elucidates the evolutionary steps underlying an adrenal androgen-dependent, human-specific ‘kill switch’ tumor suppression mechanism; and further, how it prescribes a solution to ‘normalize’ lifetime cancer risk in our species from its current aberrant 40% to the 4% that characterized primitive humans. We further argue that this prescription writ by the *lex naturalis* represents the only tenable strategy for meaningful suppression of the accelerating impact of cancer upon our species.

## Introduction

Consider these longstanding, unanswered questions in human biology. Why the extraordinary levels of circulating dehydroepiandrosterone sulfate (DHEAS) in humans – *ten thousand times higher than in mice and rats* – when its proximate metabolite (DHEA) is a potentially *irreversible* inhibitor of so crucial an enzyme as glucose-6-phosphate dehydrogenase (G6PD), the major source of the NADPH required to detoxify reactive oxygen species (ROS)? Why is the evolution of adrenarche, exclusively in humans and only our closest primate relatives, designed to flood the circulation with DHEAS just prior to the onset of adult body size? Why did humans lose the capacity to synthesize vitamin C, making our species vulnerable to the pernicious disease of scurvy? Why the evolution exclusive to our direct lineage of an alteration in the glucose-6-phosphatase (G6PC) promoter that enables inhibition of G6PD become irreversible in the presence of DHEA? And why the inactivation of uric acid oxidase (*UOX*) in our species, leading to the accumulation of extraordinary levels of circulating uric acid, precipitating the disease of gout? The uncovering of the *lex naturalis* provides a powerful answer to these longstanding, unanswered questions: Each is a component of a p53-mediated, adrenal androgen-dependent, species-specific ‘kill switch’ tumor suppression mechanism that fundamentally controls lifetime cancer risk in humans. If they had not occurred, our species could not have evolved.

Uncovering the *lex naturalis* revealed that malignant transformation represents a fundamental force opposing vertebrate speciation, and that species-specific mechanisms of tumor suppression evolved as countermeasures to suppress such opposition when speciation involved adaptive increases in body size and lifespan (Nyce 2018, 2019). Nowhere is this more apparent than in the 5000-fold increase in size from basal primates, which weighed on the order of 40 g (Gebo *et al*. 2017), to extant gorillas, which weigh as much as 200 kg. As we demonstrate here, each branchpoint in the evolution of primates that was followed by an increase in body size and lifespan was preceded by an improvement in the adrenal androgen-dependent kill switch tumor suppression mechanism.

## Primates emerged as viable species by successfully responding to the environmental conditions imposed by the PETM

Strepsirrhine (lemurs, lorises, and galagos) and Haplorrhine (tarsiers and anthropoid) primates emerged together during the Paleocene–Eocene Thermal Maximum (PETM), a period in earth’s history beginning about 56 million years ago that was characterized by a 5–8°C increase in global average temperatures. This global warming was generated by a worldwide incendiary landscape (Robson *et al*. 2014, Fung *et al*. 2018) apparently precipitated by an extraterrestrial impact (Schaller *et al*. 2016). Such a landscape would have substantially increased exposure to polycyclic aromatic hydrocarbons (PAHs), potent carcinogens formed by the incomplete combustion of organic materials. The emergence of the major subdivisions of primates during the PETM and the recent observation that extant chimpanzees preferentially scavenge at fire-combusted sites (Pruetz & Herzog 2017) suggests that primates may have required a lineage-specific tumor suppression mechanism that helped them cope with, or even enabled them to exploit, a PAH-contaminated biosphere.

The ‘kill switch’ tumor suppression mechanism that we reported is based on the extraordinarily high levels of circulating DHEAS that distinguish the primate lineage (Supplementary Fig. 1, see section on supplementary materials given at the end of this article), the very highest of which levels occur in humans, the only primate species to harness fire as a tool. Inactivation of TP53 (the human p53 gene) triggers import of DHEAS from the circulation, whereupon it is de-sulfated intracellularly to DHEA, an uncompetitive inhibitor of G6PD. That DHEA is an uncompetitive inhibitor of so critical an enzyme as G6PD has gone unappreciated for a very long time. Uncompetitive inhibition is otherwise unknown in biological systems because, in the presence of large amounts of inhibitor and substrate, it rapidly becomes irreversible (Cornish-Bowden 1986). G6PD is the cell’s major source of the NADPH required not only for reductive biosyntheses, but also to maintain redox systems in the reduced state necessary for them to neutralize reactive oxygen species (ROS) that are constantly generated in the cell. In the TP53-inactivated human cell, the levels of both DHEA inhibitor and glucose-6-phosphate (G6P) substrate rapidly rise, causing uncompetitive inhibition of G6PD to become irreversible, resulting in a catastrophic increase in ROS. Thus, the triggering of this kill switch by the inactivation of TP53 in human cells extinguishes potentially malignant cells at the single-cell stage, before they have the opportunity to evolve into the heterogeneous tumor cell populations that have made cancer incurable up to now.

If the kill switch is an effective tumor suppression system, why is lifetime cancer risk in modern humans a staggering 40%, ten times higher than has been reported for most other large, long-lived animals (Abegglen *et al*. 2015)? The human-specific tumor suppression system that evolved for the 25–30-year lifespan that has characterized humans for almost all of our existence as species (Supplementary Fig. 2). Thus, circulating DHEAS levels reach their peak at 25 years of age in humans and decline precipitously thereafter to levels inadequate to effectively trigger the kill switch. This was all that was necessary for 99.95% of our species’ existence. With lifespans that have now more than tripled, reducing DHEAS levels far below the threshold required for triggering the kill switch mechanism, modern humans are aging under the protection of only the canonical repertoire of the p53 tumor suppressor, so well-studied in the p53-knockout mouse, which by itself is effective only for vertebrate animals of small size and short lifespan – such as mice and proto primates, both of which have/had body masses of about 40 g. TP53 mutations in human tumors are thus fossils of kill switch failure, and the failure of our species-specific mechanism of tumor suppression is the cause of the 40% lifetime risk of cancer being experienced by modern humans. However, since the human-specific kill switch operates via a small molecule, DHEAS, it appears to be pharmacologically tractable. That is, it may be possible to ‘normalize’ lifetime cancer risk in our species from its current aberrant 40% to the 4% of most other large, long-lived species by pharmacologically reconstituting circulating DHEAS to peak levels and maintaining such levels throughout the modern lifespan (Supplementary Fig. 3). DHEA is not suited for this purpose and may even be dangerous, as in humans it will trigger the kill switch in unintended cells and tissues.

## The origin of primates is the origin of DHEAS secretion by the adrenal

The adrenal gland and the gonads derive from the same embryonic anlage, the adrenogonadal primordium (Keegan & Hammer 2002, Vinson 2016), and the distinguishing feature of primates – their extraordinary levels of circulating DHEAS in their adult phase – derived from the novel differentiation of the adrenal gland into a contributor to androgen synthesis. This was a dramatic evolutionary event that may have been precipitated by the PAH exposure caused by the Chicxulub asteroid impact (Kaiho & Oshima 2017), resulting in the activation in proto primates of *Alu* elements that are stimulated by DNA damage (Rudin & Thompson 2001, Hagan *et al*. 2003). This novel contribution of the adrenal gland to androgen synthesis and secretion in the primate can be considered the most primitive form of adrenarche and the singular feature that initiated the primate lineage. It was also the first step in the evolution of the human-specific kill switch tumor suppression mechanism. We will pick up our discussion now with the second step in the evolution of the kill switch tumor suppression mechanism.

## Step 2: *Alu*-mediated GLO deletion

**Figure 1:**
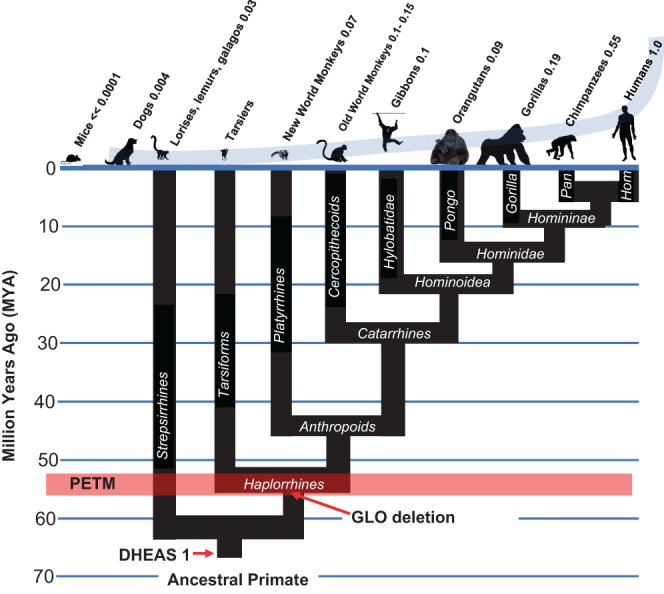
The second major step in the evolution of the human-specific kill switch tumor suppression system was the *Alu*-mediated inactivation of GLO activity in Haplorrhine primates. The numbers above each species represent their levels of circulating DHEAS, relative to human, which is set at 1.0. DHEAS levels in tarsiers, an endangered species, remain unknown. Hom, Hominini, and hominins. Hominins include those species regarded as human, directly ancestral to humans, or closely related to humans.

For uncompetitive inhibition of G6PD to become irreversible, high intracellular levels of both inhibitor (DHEA) and substrate (G6P) are necessary. While high levels of DHEA can be obtained from the extraordinary amounts of DHEAS in the circulation of 25-year-old humans, how is it possible for G6P to accumulate in TP53-affected cells when multiple enzymatic pathways exist across diverse species that act as efficient sinks for G6P? For example, the ability to synthesize vitamin C (ascorbate) is a characteristic of almost all mammals, and because it utilizes G6P as its initial substrate and is induced by elevated ROS, it would oppose kill switch function by preventing the intracellular accumulation of G6P required for uncompetitive inhibition of G6PD to become irreversible. This obstacle to kill switch function was removed by deletion of gulonolactone oxidase (GLO), the final enzyme in the synthesis of ascorbate in the genomes of Haplorrhine primates (Fig. 1). By GLO deletion, Haplorrhine primates were rendered auxotrophic for vitamin C, but this unquenchable sink for G6P was eliminated (Nyce 2019). Without this crucial early step, subsequent development of the kill switch tumor suppressor mechanism would have been impossible and humans would never have been able to harness fire as a tool. It is of interest to note that the inactivating deletion within GLO was caused by the insertion of primate-specific *Alu* transposable elements (Challem & Taylor 1998, Inai *et al*. 2003), which may themselves have been activated by PAH generated in the PETM (Rudin & Thompson 2001, Hagen *et al.* 2003, Ranjit *et al*. 2016).

## Step 3: Anthropoid primate-specific modification of glucose-6-phosphatase

**Figure 2:**
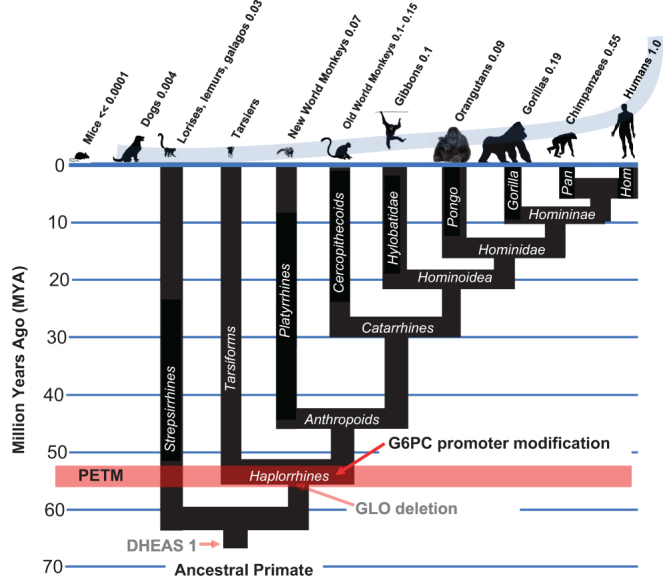
Alteration of G6PC promoter sequence motif enabled further evolution of the anthropoid primate-specific kill switch tumor suppressor system (Nyce 2018).

Glucose-6-phosphatase (G6PC) metabolizes G6P to glucose and inorganic phosphate, thereby representing another significant sink for G6P that would thwart kill switch function by preventing G6P accumulation in TP53-affected cells. G6PC activity is modulated by peroxisome proliferator-activated receptor gamma coactivator 1-alpha (PGC1alpha), which directs hepatic nuclear factor-4alpha (HNF4alpha), a member of the steroid/thyroid hormone receptor superfamily, to a specific dodecanucleotide activating regulatory site in the G6PC promoter. PGC1alpha is potently stimulated by DHEA (Yokokawa *et al*. 2015), explaining why DHEA is non-toxic to rodents – by stimulating G6PC activity, DHEA prevents accumulation of G6P in rodents, such that uncompetitive inhibition of G6PD by DHEA can never become irreversible. However, Schilling *et al*. (2008) have demonstrated that a different situation exists in humans. They identified a sequence motif in the G6PC promoter immediately downstream from the HNF4alpha-binding site that species specifically regulates PGC1alpha regulation of G6PC activity. In rodents, this sequence is ACAG, which Schilling and colleagues demonstrated, is permissive for PGC1alpha-mediated activation of G6PC activity. In humans, however, Schilling *et al*. (2008) demonstrated that this sequence motif has been changed to GAAT, which uncouples PGC1alpha from the activation of G6PD. In a species-specific manner, then, G6P can accumulate in humans in the presence of DHEA, which it cannot do in rodents. We extended this work and demonstrated that the GAAT sequence occurs throughout the anthropoid lineage, and that it is specific for this lineage (Supplementary Fig. 4). Thus, inactivation of GLO and this alteration in the sequence motif within the G6PC promoter were both required to enable G6P to accumulate to intracellular levels capable of driving uncompetitive inhibition of G6PD to irreversibility in TP53-affected cells (Fig. 2).

The canonical sequence motif at this G6PC promoter site in most vertebrate animals is GCAG (Supplementary Fig. 4). Considering the exposure to PAH experienced by primates during the PETM, it is thus of interest that the C to A and G to T mutations that created the anthropoid primate-specific GAAT sequence motif in the G6PC promoter *are the signature mutations of PAH exposure* (Kucab *et al*. 2019). This suggests that environmental exposure to PAH helped shape the evolution of the kill switch mechanism, enabling anthropoid primates to exploit the PAH-contaminated environment that they inhabited.

## Guidance by the *lex naturalis*

**Figure 3:**
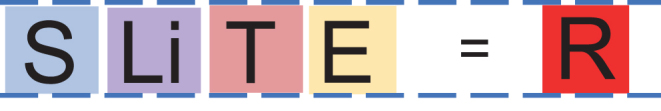
A normalized lex naturalis, representing a snapshot of a species with the dependent variables adult body size (S), lifespan (Li), species-specific mechanism of tumor suppression (T), and carcinogen exposure (E) in equilibrium to maintain lifetime cancer risk (R), at a value of about 4%.

Using empirical dynamic modeling, we have identified a relationship between body size (S), lifespan (Li), species-specific mechanism of tumor suppression (T), and exposure to carcinogens (E) resulting from the exploitation of a specific niche, such that a change in any one of these variables requires an equilibrating change in one or more of the others in order to maintain lifetime cancer risk, R, at a value of about 4%, as observed in virtually all larger, longer-lived species under natural conditions (Abegglen *et al*. 2015). As long as equilibration is possible between these dependent variables, R will remain fixed at about 4%. Figure 3 shows the *lex naturalis* equation for a species stably integrated into its environment. It represents a snapshot in time, enabling the dependent variables of the equation (S, Li, T, and E) to be shown in a normalized configuration. The *lex naturalis* equation can equally validly be drawn to illustrate the equilibrating changes that occurred during a speciation event; for example, to illustrate the improvements in species-specific tumor suppression mechanism (T), that occurred to offset increases in body size (S), and so on.

## Anthropoid primates dramatically increased in size following the combination of GLO deletion and G6PC promoter modification that improved ‘kill switch’ function

The earliest (56 MYA) primate fossils so far recovered are of a diminutive species known as *Archicebus achilles*, adult animals of which had a body length of perhaps two to three inches and a body weight estimated to be about 38g (Ni *et al*. 2013, Morse *et al*. 2019). That GLO deletion by itself was insufficient to enable significant increase in body size as a mechanism of speciation and is indicated by the fact that modern tarsiers, which were recipients of GLO deletion but not G6PC modification, remain small, averaging about 50–60 g in body mass. But within the anthropoid lineage, the basal catarrhine *Aegyptopithecus Zeuxis* (30 MYA), with an estimated body mass of 6.7 kg (Simons *et al*. 2007), demonstrates that the combination of GLO and G6PC improvements to ‘kill switch’ function enabled a remarkable 150-fold increase in body mass from basal primates such as *Archicebus achilles*.

Nevertheless, the GLO and G6PC modifications cannot account for all increases in size in the anthropoid lineage because New World monkeys (Platyrrhines) also have the combination of GLO and G6PC ‘kill switch’ improvements and yet were exceeded in body size as a lineage quite dramatically by the Old World monkeys (Cercopithecoids). For example, the critically endangered northern Muriqui (*Brachyteles hypoxanthus*), that along with the southern Muriqui (*Brachyteles arachnoides*) represent the largest New World primates, reach a body mass of 15 kg (33 lbs), are dwarfed by the largest Old World monkey, the Mandrill (*Mandrillus sphinx*), which has an average body mass of 33 kg (73 lbs). Similarly, the *Hominoidea* were enabled for much larger body size than were the Old World monkeys. According to the *lex naturalis*, these facts clearly predicted that there must be additional ‘kill switch’ improvements that distinguished cercopithecoid from platyrrhine primates and hominoids from cercopithecoids, but that we had not yet identified. We began a search to uncover them.

## Step 4: Differentiation of the *zona reticularis* in catarrhine primates, producing an intermediate form of adrenarche

**Figure 4:**
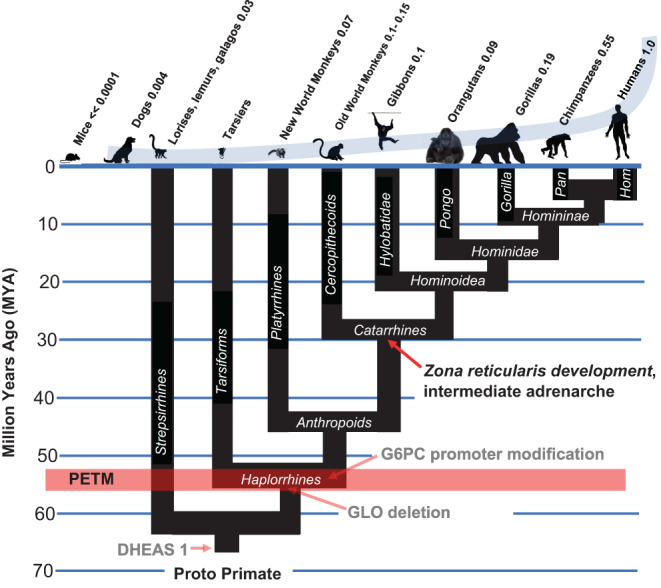
The catarrhine lineage came into existence as a result of the evolution of an intermediate form of adrenarche as an 'improvement' in their kill switch tumor suppression mechanism, enabling increases in body size that remained unavailable to Platyrrhine primates, which did not evolve this 'improvement'.

While non-primate animals derive virtually all of their DHEA from gonadal synthesis, primates have evolved a differentiation program that enables their adrenals to secrete DHEAS into the circulation. While the other tissues of the adrenal gland function throughout life – the *zona glomerulus* producing mineralocorticoids, and the *zona fasciculata* producing glucocorticoids – the *zona reticularis* emerges suddenly at about 6 years of age in humans, initiating adrenarche, the flooding of the bloodstream with prodigious amounts of DHEAS. Adrenarche occurs earlier in chimpanzees and bonobos, at 2–3 years of age (Behringer *et al*. 2012, Sabbi *et al*. 2019). Because gonadal tissue appears to regulate sexual dimorphism in humans, chimpanzees, and bonobos just as it does in other mammals, the evolutionary purpose of adrenarche has remained an enigma. DHEAS is also a neurosteroid, and its increases at adrenarche appear to parallel patterns of cortical maturation, prompting the suggestion that it plays an important role in extended brain maturation in humans (Campbell 2006). But the sudden flooding of the circulation with DHEAS, timed as it is in the developmental period immediately preceding the onset of adult body size, suggested to us that adrenarche plays an additional role as an enabling ‘improvement’ to kill switch function in humans, chimpanzees, and bonobos, focusing DHEAS secretion upon the developmental phase when it is needed (Fig. 4).

There is evidence to suggest that the origins of adrenarche exist much earlier than *Pan* and *Homo*, in the catarrhine lineage (Bernstein *et al*. 2012), where it may have also acted as an ‘improvement’ enabling increased body size, thereby distinguishing catarrhine from platyrrhine primates. Thus, a series of histologic and endocrine analyses demonstrate that catarrhine primates of both sexes have a fully functional *zona reticularis* and show an adrenarche-like secretion of DHEAS, albeit initiating much earlier than the onset of adult phase (Abbott & Bird 2009, Conley *et al*. 2011, 2018), while platyrrhine primates such as the common marmoset (*Callithrix jacchus*) do not (Pattison *et al*. 2009). The advent of an intermediate form of adrenarche in catarrhines delivers DHEAS to the circulation closer to the developmental period when it will be needed, that is, at the conclusion of the small-bodied adolescent phase and just prior to the growth period that produces adult body size. By focusing DHEAS on adult body size, the evolution of adrenarche in catarrhine primates enabled the Old World monkey lineage to explore substantial increases in body size as part of this lineage’s speciation strategy. The lack of an equivalent mechanism in New World monkeys acted as a significant constraint upon increased body size as a speciation strategy.

## What about the *hominoidea*?

The GLO deletion that had made Haplorrhine primates auxotrophic for vitamin C had also removed from the primate equation a major contributor to redox stability. Ascorbic acid is a potent antioxidant, and the loss of the ability to synthesize it *de novo* would have dramatically increased intracellular ROS concentrations (Challem & Taylor 1998). ROS are widely considered to contribute to the aging process, that is, to act as a negative pressure limiting lifespan (Munro & Pamenter 2019, Munro *et al*. 2019). Since increases in body size are inefficient if they are not accompanied by simultaneous increases in lifespan, the *lex naturalis* had pointed us in the direction of finding a replacement antioxidant to substitute for the loss of the ability to synthesize vitamin C. With this clue in mind, we turned our attention to the search for an antioxidant that replaced ascorbate in the hominoids, enabling their increase in body size.

## Step 5: Increased circulating uric acid as a critical element of kill switch evolution

One candidate molecule to replace the antioxidant activity of vitamin C is uric acid. Like vitamin C, uric acid is a potent antioxidant. In fact, with respect to one type of DNA-damaging agent (quinones) formed from the PAH produced in fire-combusted organic materials, uric acid is *superior* to vitamin C in their detoxification (Terao & Matsushita 1988). Based on *in vitro* experiments showing that uric acid was a powerful scavenger of a wide variety of ROS, in the 1980s Bruce Ames and colleagues made the suggestion that the high circulating levels of uric acid were responsible for the increases in lifespan observed in humans (also observed in other hominoids) as compared to more primitive primate species (Ames *et al*. 1981). In more recent times, a positive correlation between serum uric acid levels and lifespan in mammals has been reported (Cutler *et al*. 2019).

In our effort to investigate uric acid as a replacement for the antioxidant activity of vitamin C in post-Haplorrhine primates, we had to retrace our steps to basal Cattarhines. Thus, in addition to differentiation of the *zona reticularis* in basal catarrhine primates, there also occurred at this stage of primate evolution the first of what would become a series of changes in uric acid metabolism apparently contributing as improvements to T in the primate *lex naturalis*. Thus, Tan and colleagues investigated the kinetics and evolution of the URAT1 transport protein that is responsible for determining the amount of uric acid that is salvaged by the kidney and returned to the circulation (Tan *et al*. 2016). They determined that URAT1 of Catarrhine primates diverged sharply from the high affinity, high capacity kinetics of basal anthropoid primates (which carried into the Platyrrhines) and the low affinity, high capacity kinetics of URAT1 in the Strepsirrhines. Rather, beginning with the Catarrhines, a high affinity and *low capacity* version of URAT1 evolved that was ideal for maintaining high, constant levels of circulating uric acid. These authors noted that this evolutionary pattern suggested positive selection for increasing levels of circulating uric acid during primate evolution, but at the time could offer no suggestion on what the function of uric acid might be. Our analysis indicates that URAT1 fine tuning was the first in a series of evolutionary steps that would make uric acid, and additional mechanisms for its transport into individual cells, a critical component of the human kill switch tumor suppression mechanism.

In this regard, it turns out that hominoids are *double* knockouts. Not only was vitamin C synthesis knocked out by GLO deletion early on in primates, initiating the Haplorrhine lineage, but the enzyme responsible for the elimination of uric acid – uric acid oxidase (UOX) – was also knocked out, by a series of much later mutations (≈ 20 MYA) occurring during the Miocene epoch, creating the *hominidae* and lesser ape lineages. Inactivation of UOX – and the consequent increase in circulating uric acid that it causes – appears to have been so crucial for hominoid evolution that independent inactivation events at completely different sites in the UOX gene occurred in the hominid and gibbon lineages (Wu *et al*. 1992, Oda *et al*. 2002). The mutation that participated in the evolution of the *hominidae* occurred in codon 33 of UOX, while that which participated in the evolution of the *hylobatidae* occurred in codon 18. Still further inactivating mutations in UOX occurred in the last common ancestor of *Pan* and *Homo* species, in intron 2, and codon 187 (Kratzer *et al*. 2014).

UOX (also called uricase) is the enzyme responsible for the degradation of uric acid. Uric acid is just a steppingstone to further degradation of purines in most species, but is the endpoint in purine degradation in hominids, because of the loss of UOX gene activity in this lineage. Loss of UOX in hominids causes uric acid to reach levels as high as 400 µM in modern humans, causing a disease unique to the hominoid lineage, gout (Messerli & Bernier 2019). Inactivation of UOX – and the consequent increase in circulating uric acid that it causes – appears to be so crucial for anthropoid evolution that independent inactivation events at completely different sites in the UOX gene occurred in the hominid and gibbon lineages (Wu *et al*. 1992, Oda *et al*. 2002). Support for UOX inactivation as a component of the primate kill switch (by enabling the increase in longevity that makes increased body size efficient) comes from a recent study out of the National Institutes of Health’s National Institute on Aging. In this work, UOX^+/−^ knockout mice were observed to have large increases in circulating uric acid and an increase in longevity (Cutler *et al*. 2019).

But the transformation of URAT1 kinetics and the inactivation of UOX, together creating high circulating levels of uric acid, was not a complete solution to the ROS problem created by GLO deletion. It is not enough to have high levels of this potent antioxidant circulating in the bloodstream. It must be brought into the cell, where toxic ROS are generated. And for the kill switch to operate, such a transport of uric acid must be withheld from p53-affected cells.

## Step 6: Uric acid transport into the cell put under p53 control

Only hominoid primates have high uric acid blood levels. Add to this the fact that more than 90% of uric acid is reabsorbed by the kidneys instead of being excreted, and it becomes clear that uric acid has been converted from a waste product (as it is in most other species) to something useful in hominoid primates. As noted, the usefulness of uric acid may be that it enabled this lineage to overcome the increase in ROS caused by loss of the ability to synthesize vitamin C. But uric acid in the circulation cannot neutralize ROS inside a cell. For this, active transport mediated by a cell-surface protein is required, in the same way that it is for DHEAS in the circulation.

**Figure 5:**
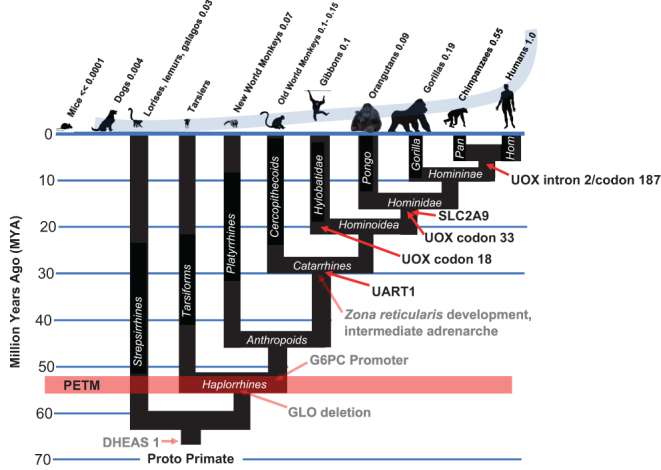
Increasing the levels of circulating uric acid, and putting the uric acid transporter under p53 control, dramatically improved kill switch function, enabling the remarkable increases in size of the *Hominoidea*. The existence of these components of the kill switch tumor suppression system was predicted by the *lex naturalis*. Adrenarche 2 refers to the intermediate form of adrenarche observed in catarrhine primates.

Uric acid is imported into the cell by a widely expressed cell-surface protein called SLC2A9 (https://www.proteinatlas.org/ENSG00000109667-SLC2A9/tissue). Itahana *et al.* (2015) have made the important discovery that SLC2A9 is under p53 control in hominids. These authors demonstrated that in human cells undergoing oxidative stress, SLC2A9 expression increased in a p53-dependent manner – if they knocked out p53 expression, SLC2A9 expression was eliminated as well, and uric acid was not transported from the circulation to the interior of the cell. They showed that active expression of SLC2A9 in TP53-competent cells reduced ROS and protected against DNA damage and cell death. Putting SLC2A9 under positive TP53 control represents an additional critical event in the evolution of the human-specific kill switch tumor suppression mechanism. Thus, all normally functioning cells with active TP53 are protected from intracellular ROS by the import of antioxidant uric acid, as needed. In effect, uric acid replaced vitamin C as an antioxidant, but in a manner that was, unlike vitamin C, completely dependent upon there being active TP53 in the cell. This critical next step in hominid evolution also potentiated the selective killing power of the primate-specific kill switch tumor suppression mechanism, such that TP53 inactivation not only triggered the active import of circulating DHEAS into the cell, but now switched *off* the import of antioxidant uric acid as well. In the p53-affected hominid cell then, DHEAS is imported and de-sulfated to DHEA, enabling irreversible inhibition of G6PD, thereby elevating ROS to levels that are catastrophic for the cell. Simultaneously, the import of uric acid that might oppose cell death by neutralizing ROS is switched *off* by p53 inactivation (Fig. 5).

## Step 7: Further improvements in T required by meat consumption

**Figure 6:**
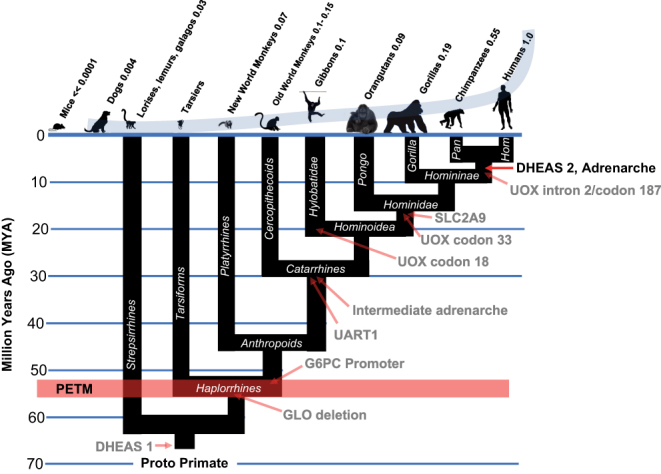
The last common ancestor of the chimpanzee and hominin lineages consumed meat, increasing E, and thereby requiring an equilibrating alteration in T, which consisted of a three-fold increase in circulating levels of DHEAS and the evolution of a complete form of adrenarche.

The last common ancestor of chimpanzees and hominins distinguished itself from the gorilla and the orangutan by including red (mammalian) meat in its regular diet. The International Agency for Research on Cancer (IARC) has classified red meat as ‘probably carcinogenic,’ and heat-processed meat as ‘carcinogenic’ (https://www.iarc.fr/wp-content/uploads/2018/07/pr240_E.pdf). Follow-on studies have provided additional evidence demonstrating the carcinogenicity of red meat in a variety of human cancers (Dialo *et al*. 2018). In a recent large study out of the National Cancer Institute which followed almost a half million people with a median follow-up of 15.5 years, substitution of plant protein for red meat protein was found to substantially reduce cancer risk (Liao *et al*. 2019). The addition of red meat in the diet of the last common ancestor of chimpanzees and hominins thus increased carcinogen exposure, E, which was countered by an offsetting increase (almost three-fold) in circulating DHEAS, and by precisely focusing adrenarche to precede the onset of adult body size (Fig. 6). Mechanisms by which circulating DHEAS were increased during the evolution of the *homininae* are likely to have involved transcription factor-binding site changes (Bernstein *et al*. 2012). The mechanisms by which adrenarche focuses DHEAS secretion in a development-specific manner is under intensive investigation (Thomas *et al*. 2015, Udhane & Fluck 2016, Maizoub & Topor 2018).

## The *lex naturalis* explains important features of primate evolution

**Figure 7:**
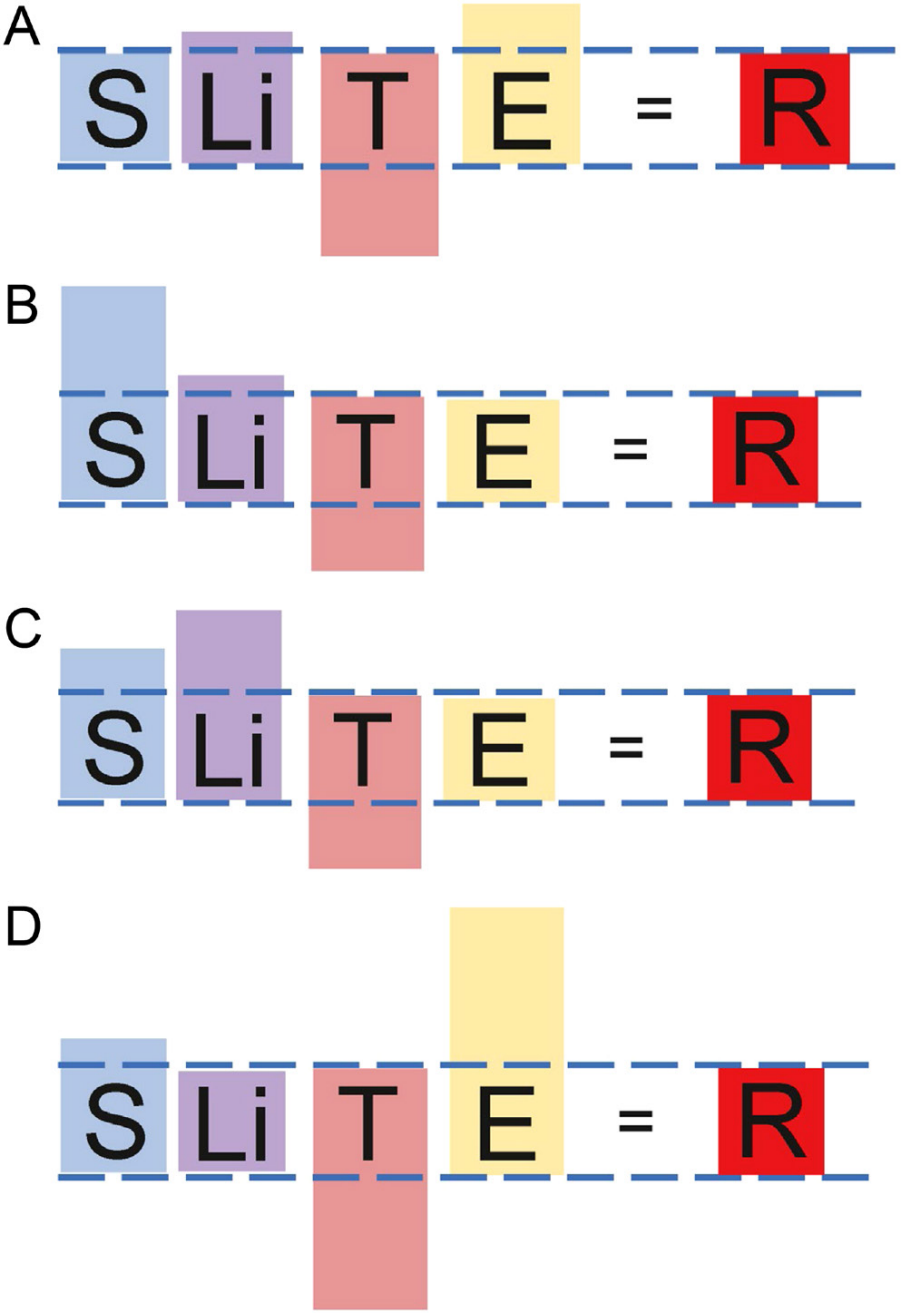
*Lex naturalis* equations for some hominid species. (A) *Lex naturalis* equation for the last common ancestor of chimpanzees and hominins, distinguished from other hominids by the consumption of meat, increasing E, necessitating an equilibrating increase in T. (B) *Lex naturalis* equation for gorillas, which maintained very low E by consumption of a vegetarian diet, enabling dramatic increase in body size, S. (C) *Lex naturalis* equation for orangutans, also vegetarian. In contrast to gorillas, orangutans emphasized lifespan over body size. (D) *Lex naturalis* equation for primitive *Homo sapiens*, who experienced dramatic increase in E as a result of the harnessing of fire and consumption of heat-processed meat. Although constrained, body size continually increased from *Homo habilis* to *Homo erectus* to *Homo sapiens* as human skills with fire, tools, and weapons increased consumption of meat, especially heat-processed meat, continuously increasing E during human evolution, requiring continuous improvements in T. Such improvements in T appear to have been accomplished by increasing circulating DHEAS.

The *lex naturalis* teaches that increases in body size and lifespan cannot occur in vertebrate animals without corresponding improvements in species-specific mechanism of tumor suppression (T). Increases in carcinogen exposure also require improvements in T for the species to remain stable, that is, with R maintained within limits required by the *lex naturalis*. Thus, chimpanzees and early hominins (e.g. *Australopithecus afarensis*) could not significantly increase size or lifespan, even though they had improved their species-specific mechanism of tumor suppression by augmenting their circulating DHEAS, because they evolved from an ancestor that had substantially increased E by consuming red meat (Fig. 7A).

Gorillas and orangutans, on the other hand, preserved a very low E by maintaining a vegetarian diet. Accordingly, they were able to increase both body size and lifespan. Whereas gorillas emphasized body size in their solution of the *lex naturalis* (Fig. 7B), orangutans emphasized lifespan (Fig. 7C). Gorillas also doubled their levels of circulating DHEAS (compared to ancestral *Hominidae*) to enable their dramatic increase in body size. In fact, increasing body size to expand niche exploitation reached remarkable levels overall in the primate lineage. The relative increase in size from primitive Haplorrhines, which weighed just a few tens of grams (Morse *et al*. 2019), to the gorilla, which can weigh 200 kg, exceeded even the increase in size from *Pakicetus* (the wolf-sized land animal that would give rise to whales) to *Balaenoptera musculus*, the blue whale, the largest creature ever to have lived on this planet. Clearly, the dependent variables of the *lex naturalis* required continuous equilibration during such expansion of the primate lineage, with S, body size, particularly competing with E, exploitation of a carcinogenic niche, to acquire the benefit of each improvement in T, species-specific mechanism of tumor suppression.

## Step 8: The pyrophilic primate: Selection for high levels of circulating DHEAS

The earliest archeological evidence for use of fire by our immediate ancestor, *Homo erectus*, occurs at sites in East Africa, including Cheswanja, near Lake Baringo, Koobi Fora, and Olorgesailie in Kenya, all dated to about 1.5 MYA (Gowlett 2016). Some authors attribute even earlier *Homo* species as adapting to fire prone environments 2 to 3 million years ago in tropical Africa, in fact becoming dependent upon natural fire to survive the extreme and rapid fluctuations between closed canopy forests, woodland, and grasslands that occurred at this time (Parker *et al*. 2016). As we have noted, humans are the only primate to have harnessed fire as a tool, although other primates such as the chimpanzee (and even non-primates, such as the hawk) capitalize upon natural fires as excellent sites at which to forage (Pruetz & Herzog 2017). The harnessing of fire led to PAH exposure levels in unventilated human habitats that are far in excess to anything that we can imagine in the modern world. PAH are potent mutagens, carcinogens, and teratogens and therefore critically increase E, at the expense of other of the dependent variables of the *lex naturalis*, particularly S and Li. Although a steady increase in body size did occur from the Australopithecines to and through *Homo* species, the intense increase in E experienced by the *Homo* lineage as a result of the harnessing of fire set significant constraints upon such increases. Despite such constraints, steady increases in body size occurred as *Homo* species evolved, clearly adaptive in an environment dominated as it was by large carnivores and intertribal warfare. But to achieve these increases in body size, humans required still further adaptive improvements to T, and they acquired those improvements by undergoing selection for members of the species who had the highest levels of circulating DHEAS and by continuing to accept constraints upon lifespan, as compared to gorillas and especially to orangutans (Fig. 7D).

Intelligence in primates also gives the appearance of being related to circulating levels of DHEAS, although the totality of factors involved in this aspect of primate evolution are clearly manifold. DHEAS has been shown to play a critical role in human cognition and memory (Nguyen *et al*. 2018) and may have been doing so throughout primate evolution. With increasing intelligence in human species, the ability to fashion tools and weapons for hunting and butchering increased meat consumption still further, which many believe may have fueled encephalization (Richards 2002, Pontzer *et al*. 2016). Evidence for the use of stone tools to cut and strip meat from bones occurred by at least 2.6 MYA, as demonstrated by archeological sites that link stone tools with cut-marked bones (Wrangham *et al*. 1999, Plummer 2004, Pobiner *et al*. 2008, Merritt 2017). Eating energy-dense meat and the harnessing of fire together dramatically increased E in *Homo* species, which was offset by increased levels of circulating DHEAS, and constraint upon increase in size, and even more constraint upon lifespan, while brain size and function increased without similar constraint.

## Improvements in kill switch tumor suppression occur at every major branch point in primate evolution that is followed by increased body size and lifespan

**Figure 8:**
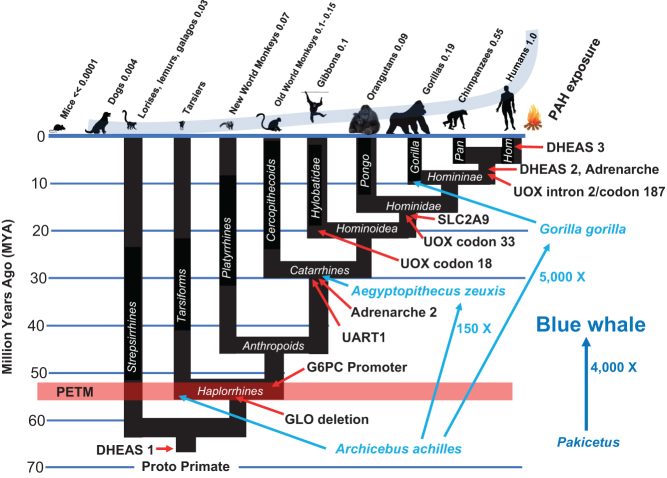
Improvements in the primate-specific kill switch tumor suppression mechanism occur at every branch point in the tree of primate evolution in which adaptive increases increases in body size and lifespan, or increased exposure to a carcinogenic environment, subsequently arise. Increases in size during primate speciation reached an astonishing level, surpassing even those from *Pakicetus*, the wolf-sized land mammal that gave rise to the whales, to *Balaenoptera musculus*, the Blue whale. Int. adrenarche, intermediate adrenarche.

Our central discovery is that malignant transformation represents a limiting force opposing vertebrate speciation, and that species-specific mechanisms of tumor suppression evolved as a counterforce to overcome such opposition. Species-specific mechanisms of tumor suppression are thus fundamental elements of vertebrate speciation without which adaptive increases in body size and lifespan could not occur. *Improvements in the primate-specific kill switch tumor suppression mechanism occur at every branch point in the tree of primate evolution in which adaptive increases in body size and lifespan, or increased exposure to a carcinogenic environment, subsequently arise* (Fig. 8).

## The dis-equilibrated *lex naturalis* equation of modern humans

**Figure 9:**
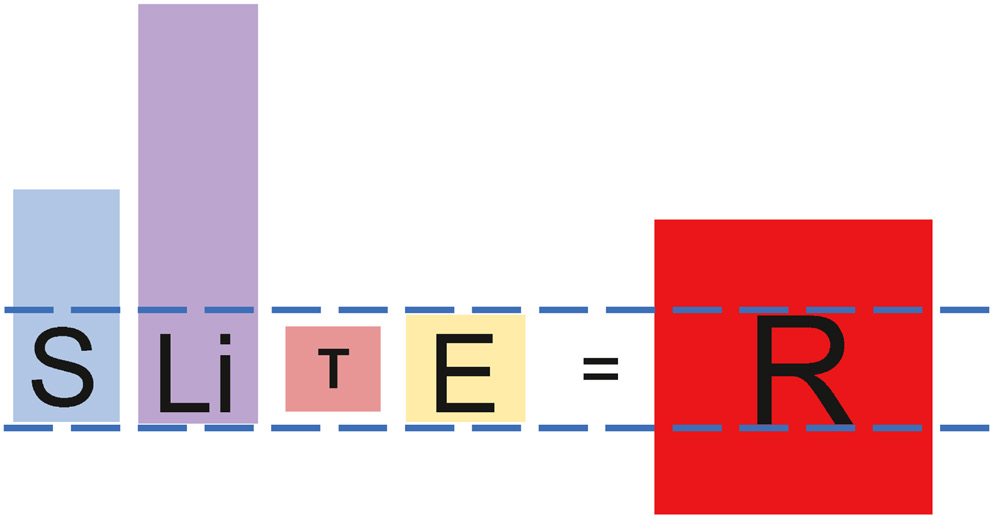
*Lex naturalis* equation for modern humans. Due to the modern economy, body size, S, has more than doubled compared to primitive members of our species. Due to modern medicine and public healthcare systems, Lifespan, Li, has more than tripled. This has left aging humans without a functioning species-specific mechanism of tumor suppression, T, because circulating DHEAS precipitously declines after the age of 25, in keeping with the 25–30 year lifespans of primitive humans. Consequently, due to the impossibility of equilibration among the dependent variables, R has dramatically increased.

The *lex naturalis* explains the current increase in R that has occurred in modern humans. As illustrated in Fig. 9, R in modern humans is 40%, tenfold higher than the 4% of other large, long-lived animals, as well as primitive members of our own species. This tenfold increase in R has been driven by a twofold increase in adult body size, S, in modern humans as compared to primitive members of our species, made possible by the modern economy; and a more than threefold increase in lifespan, Li, made possible by modern medicine and public health measures. T, the human-specific mechanism of tumor suppression, has been rendered ineffectual in modern humans by the precipitous decline in circulating DHEAS that occurs after the primitive lifespan has been surpassed (Supplementary Fig. 2). Except for cigarette smokers and occupational exposure to carcinogens, E in modern humans may be equivalent to or possibly even reduced compared to what it was in PAH-saturated primitive humans surviving in their smoke-filled habitats. In terms of the *lex naturalis*, changes in the dependent variables of S, Li, and T have been so extreme that no equilibration between them has been possible, resulting in a dramatic increase in R, the effects of which our species is now experiencing.

## The *lex naturalis’* prescription for ‘normalizing’ human cancer risk

**Figure 10:**
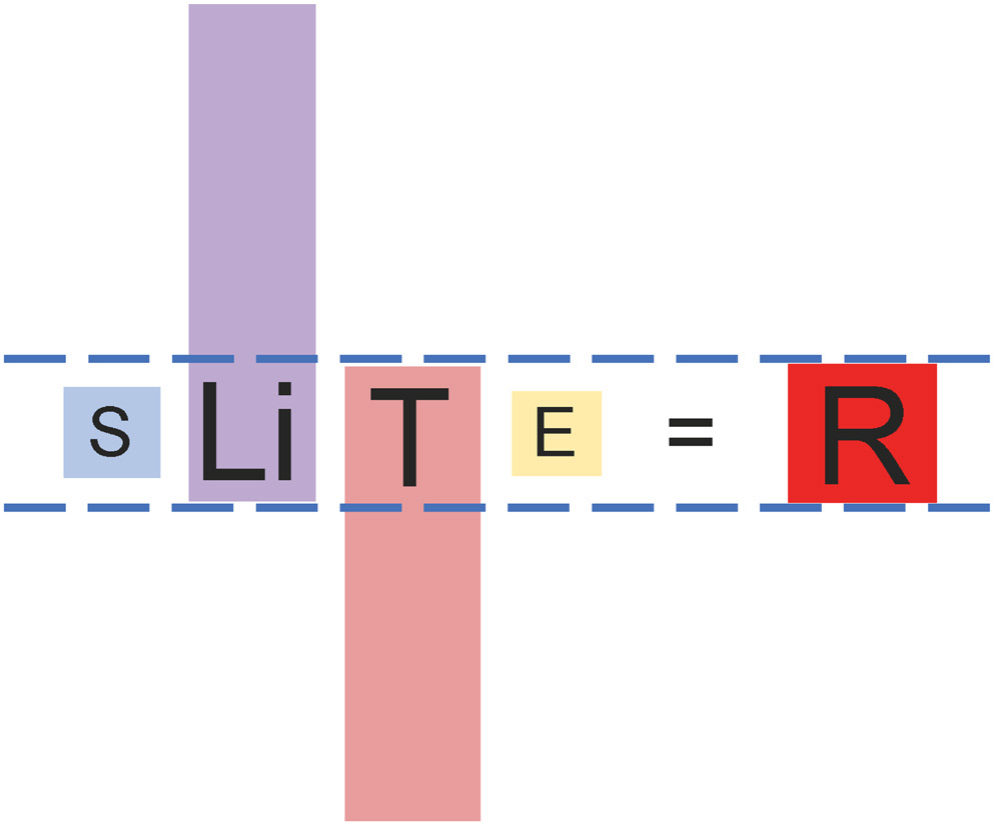
The *lex naturalis* prescription to ‘normalize’ lifetime cancer risk, R, in our species.

The past five decades of cancer research have focused on finding a ‘cure’ for cancer or at least producing treatments that increase survival time. Great strides were made over these decades, but survival in cancer patients appears now to have reached an asymptotic boundary beyond which any further significant improvement may be impossible (Supplementary Fig. 5). Certainly a ‘cure’ now appears impossible due to the vast heterogeneity displayed by tumor cell populations. A different approach suggested by the *lex naturalis* is the ‘normalization’ of lifetime cancer risk in our species from its current aberrant 40% to the 4% that characterizes other vertebrates in the wild, including our own in the primitive setting in which it evolved (Fig. 10).

## T, species-specific mechanism of tumor suppression

Unlike the species-specific mechanisms of tumor suppression observed in other species, which are genetic (Abegglen *et al*. 2015, Sulak *et al.* 2016, Vazquez *et al*. 2018), the human-specific tumor suppression mechanism operates via a small molecule, DHEAS, and is therefore pharmacologically tractable. Thus, we can pharmacologically reconstitute peak levels of circulating DHEAS as soon as they begin to decline and maintain these peak levels throughout the modern lifespan. By this measure, the *lex naturalis* predicts that we may be able to restore a lifetime cancer risk approaching 4% in modern humans despite even further increases in lifespan.

## E, carcinogen exposure

We know from occupational exposures, and from the lung cancer epidemic that followed the introduction of tobacco products after the First World War, and its decline after the Surgeon General forced tobacco companies to put warnings on cigarette packaging, that hotspots of carcinogen exposure exist and drive E to exceedingly high levels in exposed individuals. We should therefore continue, with increased vigor, to eliminate all human exposure to carcinogens, in order to reduce E to as low a figure as possible – including the carcinogenic potential of red meat. The *lex naturalis* suggests the possibility that the carcinogenicity of red meat may derive not from carcinogens that it contains, but rather from its ability to influence adult body size when consumed during developmental growth periods that occur prior to puberty.

## S, adult body size

As demonstrated in Supplementary Fig. 6, men have a significantly higher lifetime cancer risk than women do. The average man is also significantly taller than the average woman, a fact which the *lex naturalis* suggests may be responsible for some, perhaps most of the increased lifetime cancer risk for men. There is additional evidence implicating increasing height with increasing lifetime cancer risk in our species. For example, the recent ‘million women study,’ which followed 1,297,124 women for a median time of 9.4 years each, reported an overall 16% increase in cancer risk for every 10 cm (4 inches) in height above average (Green *et al*. 2011). This association of increased cancer risk with increased height has been confirmed by additional studies performed in 144,701 women (median follow-up, 12 years) (Kabat *et al*. 2013), and in 310,000 male and female UK Biobank participants (Ong *et al*. 2018). Thus, the taller a human is, the greater their lifetime cancer risk, in both sexes. Conversely, the shorter a human is, the lower their lifetime cancer risk, in both sexes. At this shorter end of the height spectrum, studies of dwarf humans with Laron Syndrome – one of which studies lasted 57 years – demonstrated a near total absence of cancer in these long-lived, small-bodied humans (Janecka *et al*. 2016, Laron & Kauli 2016).

## According to the *lex naturalis*, a short child is a well-adapted child

Correlations between nutritional status – associated with socioeconomic status – and adult height have led to a nutrition philosophy in which developmental success is measured as a function of increased stature (Perkins *et al.* 2016, Park *et al*. 2019). As evidence of this, consider the physical exams that we subject our children to during their development. All of the height indices by which developmental ‘progress’ is measured by pediatricians are designed to show where our children stand with respect to the mean, and a pediatrician is apt to express concern if a child is substantially below that mean (‘height-for-age’ *Z* score) and to do the opposite if the child is well above. This sets up a positive feedback system which drives toward greater and greater height in our species. Increased stature was immensely important in the primitive human landscape, dominated as it was by predation by large carnivores, and by intertribal warfare, and in which humans lived short, accelerated lives. But outside of sporting arenas, increased stature is not useful in the modern world and is working against us as a species. Our continued, misplaced preoccupation with increasing stature is one of the principal dis-equilibriums in the *lex naturalis* of modern humans. How might we begin to correct this disequilibrium? Two growth periods have been found to be important in determining adult height. The first of these occurs from conception to about 2 years of age. The second occurs from 2 years up to the onset of adrenarche. Overnutrition that stimulates *Z* during these growth phases may be just as bad for our children, in the long run, as malnutrition. If we can find ways to reduce adult height (without negatively affecting mentation) by adjusting our nutritional goals for our children during these critical growth periods, re-equilibration of adult body size, S, combined with DHEAS reconstitution of the ‘kill switch,’ may enable rapid normalization of lifetime cancer risk, despite even further increases in lifespan, Li.

## Li, Lifespan

Lifespan is not a dependent variable that humans are likely to adjust in a downward direction in order to equilibrate R, lifetime cancer risk – although certainly reducing Li to that of primitive humans would do so. It remains to be determined what the possible human lifespan is, but with proper equilibration of the *lex naturalis*, it would not be surprising to see it rise significantly above its current value. Of course, such long life would only be desirable if we could live it in relative health, absent of many of the diseases of old age which now plague us. The growing literature on the effects of DHEAS in cognition, memory, and other aspects of brain function suggest the possibility that reconstitution of T and reduction in S may have positive effects beyond the normalization of R.

## A new strategy: ‘Normalization’ of lifetime cancer risk

The *lex naturalis* predicts that we can ‘normalize’ human lifetime cancer risk by pharmacologically reconstituting our kill switch tumor suppression system. It would be useful to achieve some verification of the ‘normalization’ hypothesis in an animal model, but this is ruled out by the *lex naturalis*: *the existence of species-specific mechanisms of tumor suppression prevent one vertebrate species from acting as a valid model system for cancer in another vertebrate species* (Supplementary Fig. 7). No other species evolved in the presence of such high levels of circulating DHEAS, rendering meaningless preclinical toxicology and efficacy studies performed in non-human species.

The practical question that we are thus faced with is that the potential to normalize human lifetime cancer risk from its current 40% to the 4% observed in other large, long-lived animals is a testable hypothesis, but only in humans. As we have noted, the *lex naturalis* powerfully explains an array of longstanding questions in human biology. *Why the extraordinary levels of circulating DHEAS in humans, when DHEAS’s proximate metabolite, DHEA, is an uncompetitive, potentially irreversible inhibitor of so crucial an enzyme as G6PD? Why the evolution of adrenarche, a developmental phase that floods the human circulation with DHEAS immediately preceding the onset of adult body size? Why the loss of GLO activity, leading to vitamin C auxotrophy, and the potential to develop scurvy? Why the evolution of a completely anthropoid primate-specific GAAT sequence motif in the G6PC promoter that enables inhibition of G6PD to become irreversible in the presence of DHEAAnd why UOX deletion, leading to accumulation of circulating uric acid, and the disease of gout?* It is extremely unlikely that so many longstanding, unresolved questions in primate evolution all intersect at kill switch function, and obtain compelling explanation by such intersection, if that is not where they derive their evolutionary significance. We therefore argue that the kill switch we describe represents our species’ primary defense against cancer, with a probability approaching 1, despite the impossibility of pre-confirmation in animal models. Similarly, the *lex naturalis* has powerful explanatory power not only for primate mechanisms of tumor suppression, but across the spectrum of vertebrates speciation (Supplementary Figs 7, 8, 9, 10, 11, and 12).

The IARC projects that by 2040 the number of new cancer cases will reach almost 30 million per year and cancer deaths nearly 17 million per year, and has warned that these are not numbers that we have any chance of treating our way out of (IARC 2018, 2019). The possibility of normalizing lifetime cancer risk in our species by addressing the disequilibrium in the modern *lex naturalis* offers an alternative, prevention strategy that has the potential to substantially mitigate the accelerating impact of cancer upon our species. Since DHEAS is a natural constituent of the human body, and is not a potent uncompetitive inhibitor of G6PD in the manner of DHEA, it is hard to imagine a safer clinical trial than the reconstitution of DHEAS to their peak levels and the maintenance of such levels throughout the modern lifespan. While it might be argued that maintenance of DHEAS at such high levels could aggravate hormone-sensitive tumors, such as breast and prostate cancers, the point of the kill switch tumor suppression mechanism is that it would prevent such tumors from developing in the first place. Considering the apparent safety of DHEAS, and the dystopian cancer numbers that we are approaching guided by our current cancer research paradigms, clinical studies to test the normalization hypothesis should become a focus of discussion at the National Cancer Institute and the IARC – the only two agencies capable of conducting clinical investigations on the scale that will be required. Direct testing in humans will require breaking with a paradigm that formed in the absence of knowledge of the *lex naturalis* – that anti-cancer strategies must be pre-confirmed in animal models. But in view of the *lex naturalis*, abandonment of this paradigm appears to be the only prudent way forward.

## Supplementary Material

Supplementary Section 1

## Declaration of interest

J W N has pending patents related to kill switch maintenance, patents he is willing to transfer to the NCI and/or IARC under certain conditions.

## Funding

This research did not receive any specific grant from any funding agency in the public, commercial, or not-for-profit sector.
