# Supplementary material for: A lex naturalis delineates components of a human-specific, adrenal androgen-dependent, p53-mediated ‘kill switch’ tumor suppression mechanism
Source: Endocr Relat Cancer. 2019 Dec 9;27(2):R51–65. doi: 10.1530/ERC-19-0382 (PMC6993206; doi:10.1530/ERC-19-0382)
Supplement: Supplementary Section 1 [file supplementary_figure_1.pdf]

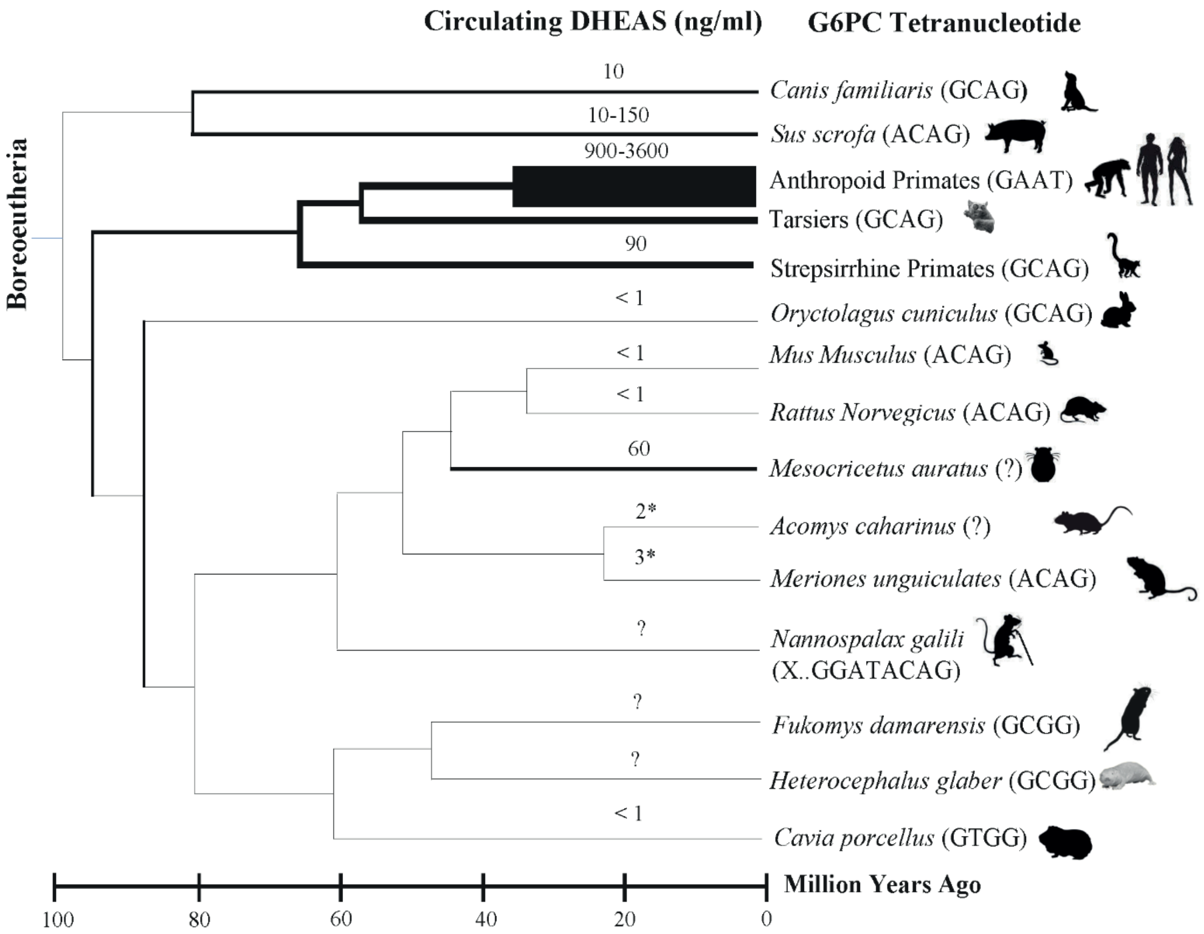

S1. Primates are distinguished by their extraordinarily high levels of circulating DHEAS

Previously published in: https://www.ncbi.nlm.nih.gov/pmc/articles/PMC6106910/

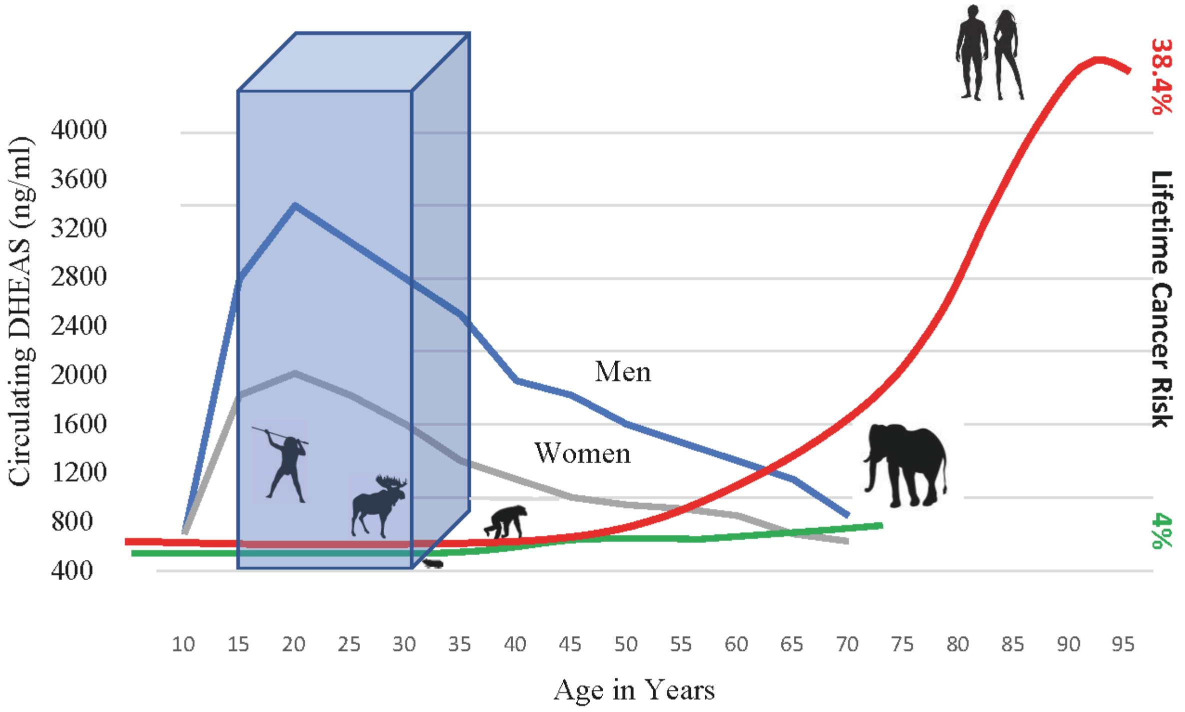

S2. The human-specific “kill switch” tumor suppression mechanism evolved for the 25-30 year lifespans of primitive humans, and because circulating levels of DHEAS precipitously decline thereafter (blue and grey lines), fails in long lived modern humans. Previously published in: https://www.ncbi.nlm.nih.gov/pmc/articles/PMC6106910/

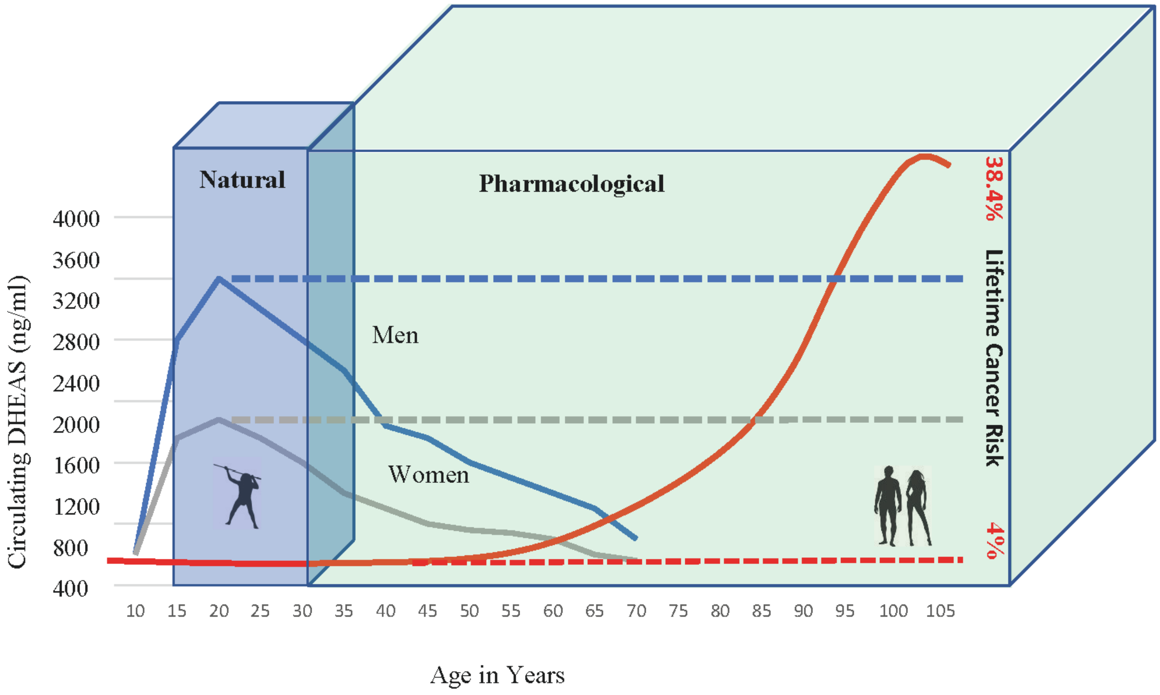

S3. Because the human-specific “kill switch” tumor suppression mechanism is based on a small molecule, DHEAS, it is pharmacologically tractable. By maintaining peak levels throughout the modern lifespan, it may be possible to reconstitute kill switch function, and “normalization” of lifetime cancer risk in humans from its current aberrant 40% to the 4% of other long-lived, large animals.

Previously published in: https://www.ncbi.nlm.nih.gov/pmc/articles/PMC6106910/

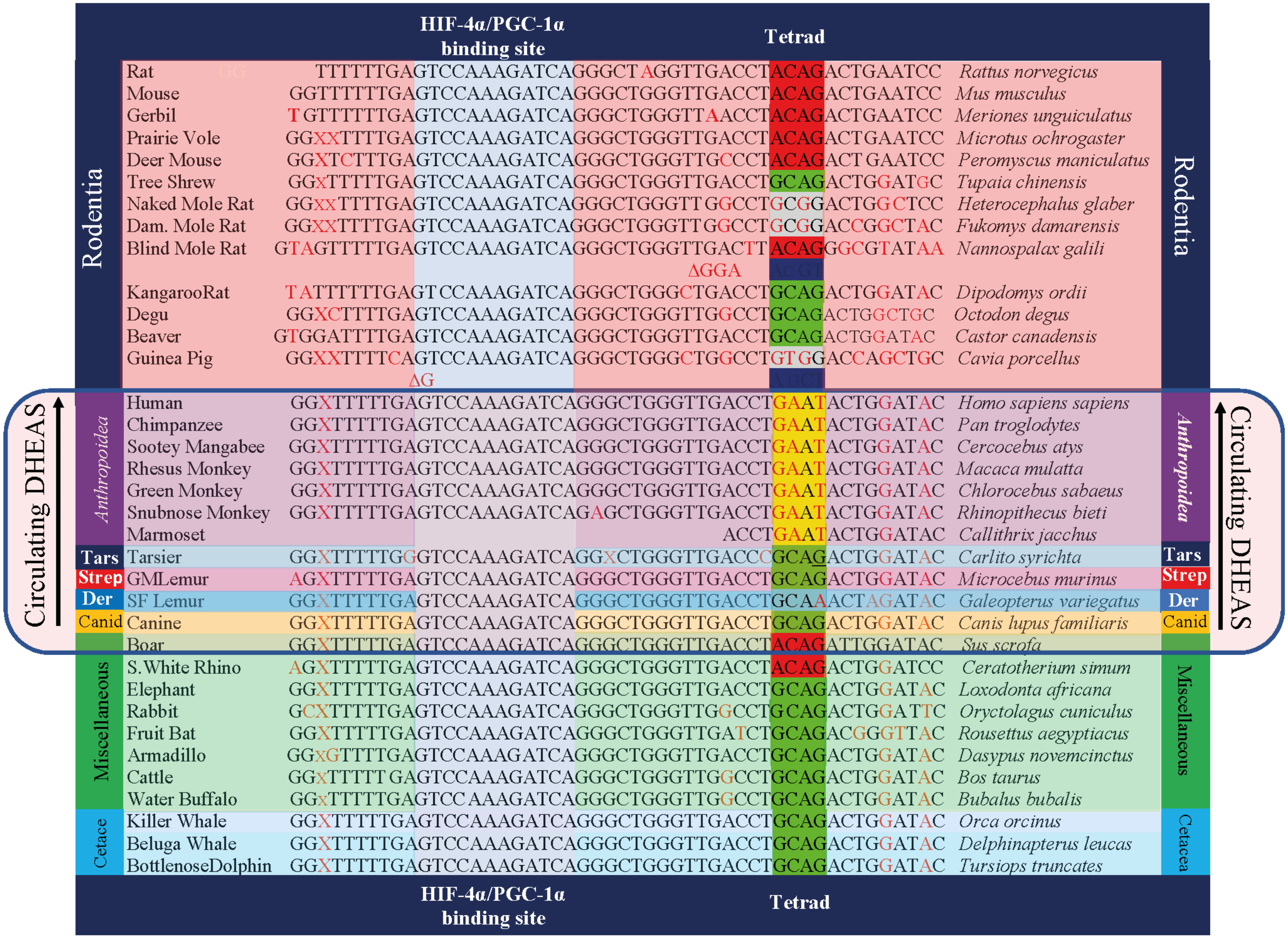

Figure S4. The GAAT tetrad sequence motif downstream from the HIF-4α/PGC-1α activating binding site in the G6PC promoter is unique to anthropoid primates, and enables G6P to accumulate in TP53-affected cells. Previously published in: https://www.ncbi.nlm.nih.gov/pmc/articles/PMC6106910/

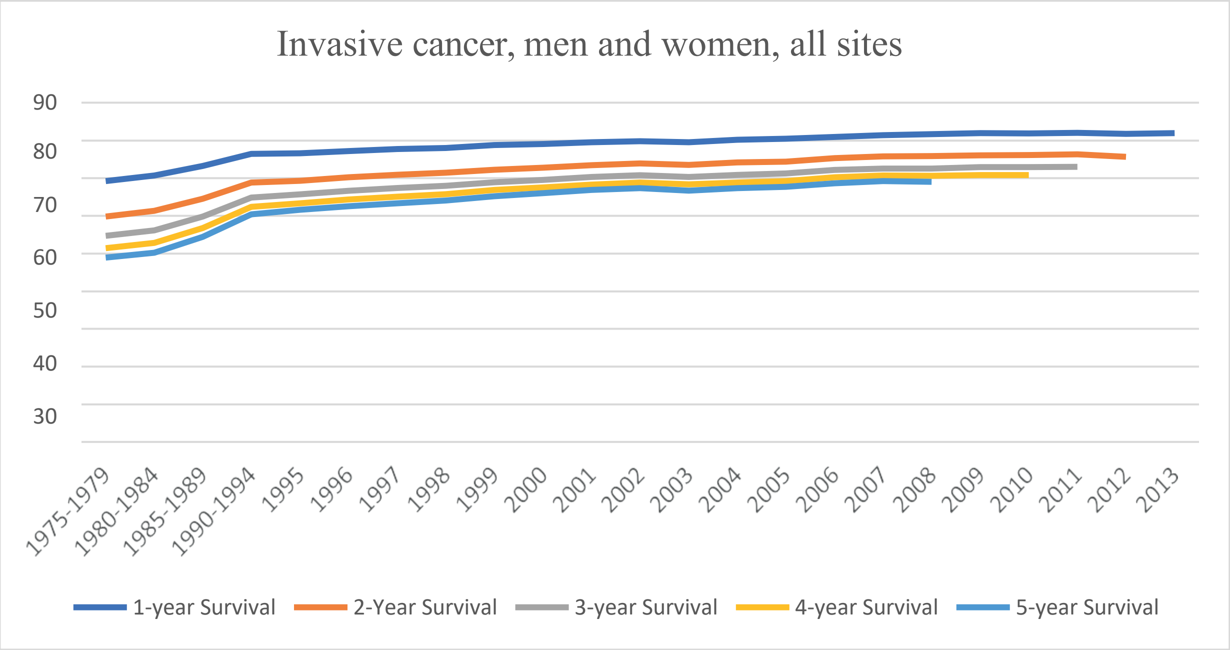

Figure S5. National Cancer Institute statistics demonstrate that patient survival at all time points are at an asymptotic limit beyond which significant further progress may be impossible. Furthermore, these data show that 2-year survival has improved only 7% over the past 27 years. Data from NCI SEER Cancer Statistics Review (CSR) 1975-2014. Updated June 28, 2017. https://surveillance.cancer.gov/statistics/types/survival.html

Previously published in: https://www.ncbi.nlm.nih.gov/pmc/articles/PMC6106910/

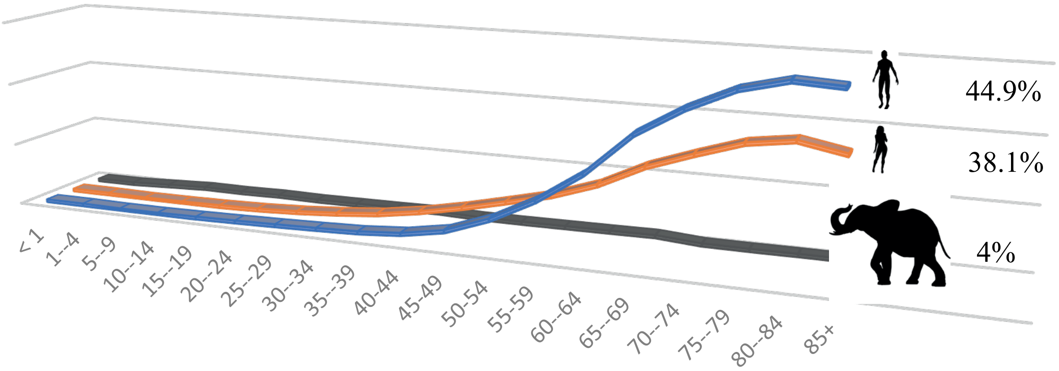

Figure S6. Cancer risk is an order of magnitude higher in humans than it is in other long-lived species, here represented by the elephant. Cancer risk is also significantly higher in males as compared to females of our species. Data from NCI SEER Cancer Statistics Review (CSR) 1975-2014. Updated June 28, 2017. https://surveillance.cancer.gov/statistics/types/survival.html; and Abegglen *et al*.[9]

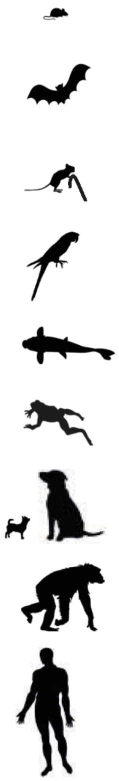

In vertebrate animals, species-specific mechanisms of tumor suppression *enable* species-specific adaptations such as body size and lifespan that, in turn, enable maximized exploitation of a species' specific niche. Thus, unique mechanisms of tumor suppression *distinguish* vertebrate species. In-so-far as neoplastic transformation is concerned, *species-specific mechanisms of tumor suppression prevent one vertebrate species from serving as a valid model system for another*. This largely unrecognized element of speciation undermines decades of cancer research data, using murine species, that presumed universal mechanisms of tumor suppression, independent of species…

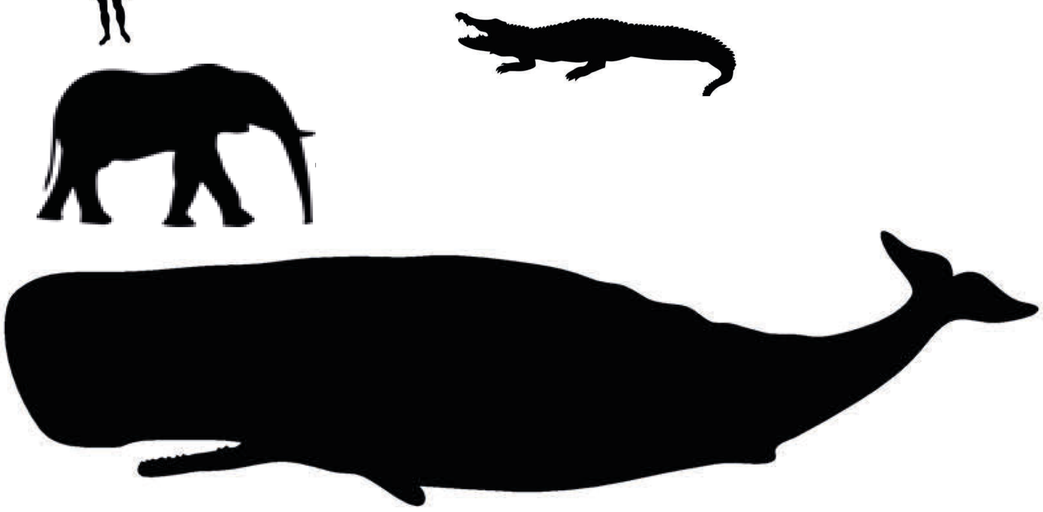

Figure S7. Previously published in: https://doi.org/10.1530/ERC-18-0468

The *lex naturalis* in large vs. small dog breeds

If our various breeds of dogs had evolved in the wild, they would have separated into different species, with differing tumor suppression strategies to go along with their different environmental adaptations. Our manipulation of the canine *lex naturalis* equation away from that of the grey wolf by breeding to enhance specific traits provided some advantages to our smallest dogs, at least in terms of lifespan. Reducing their body size violated the *lex naturalis*, which caused an equilibrating increase in lifespan. But when we selected for large increases in body size, as for example in the Irish Wolfhound, without providing such dogs with an improved tumor suppression system the way natural selection would have done, this violation of the *lex naturalis* caused the opposite effect— an equilibrating decrease in lifespan. And when decreasing lifespan reached the point that it was no longer tenable because of the decrease in efficiency it caused, the *lex naturalis* was enforced by an equilibrating increase in lifetime cancer risk. Thus, our giant dog breeds live short, accelerated lives, with a dramatically increased lifetime cancer risk.[1]

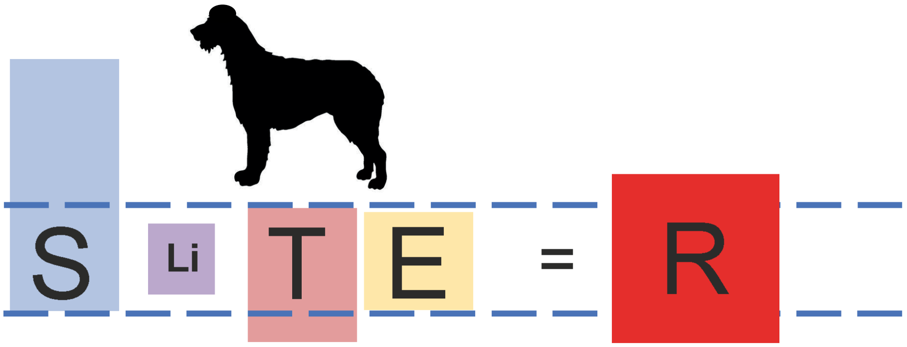

S8. Pictorial representation of the *lex naturalis* equation for large dogs

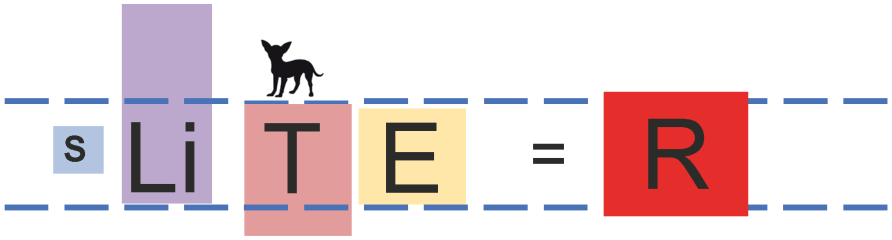

S9. Pictorial representation of the *lex naturalis* equation for small dogs

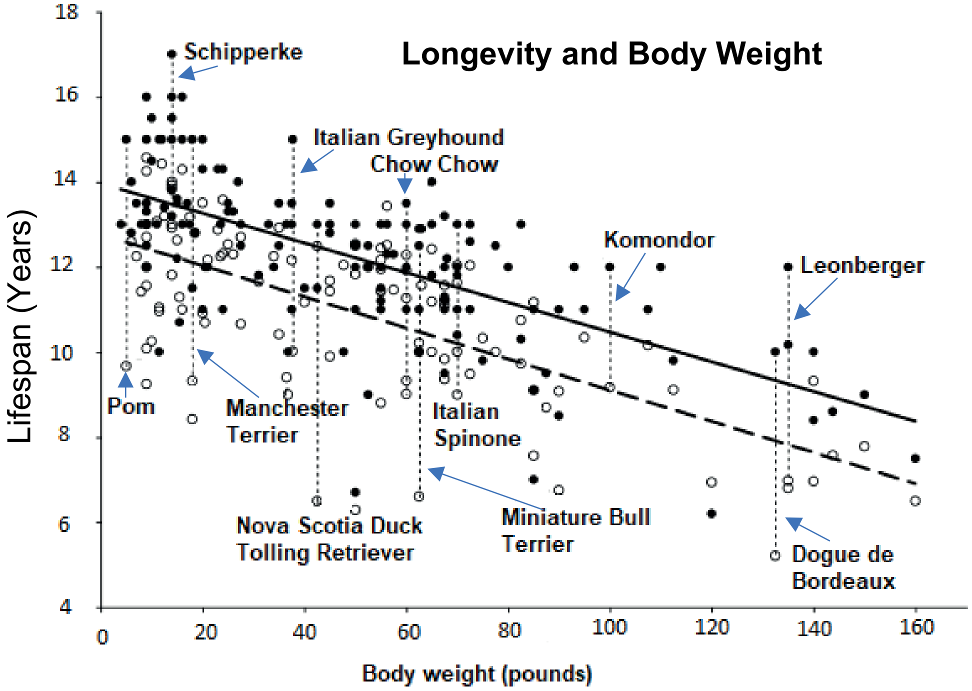

Figure S10 Relationship between longevity and body weight in dogs. Figure 4 from Jones *et al.* 2008[2]

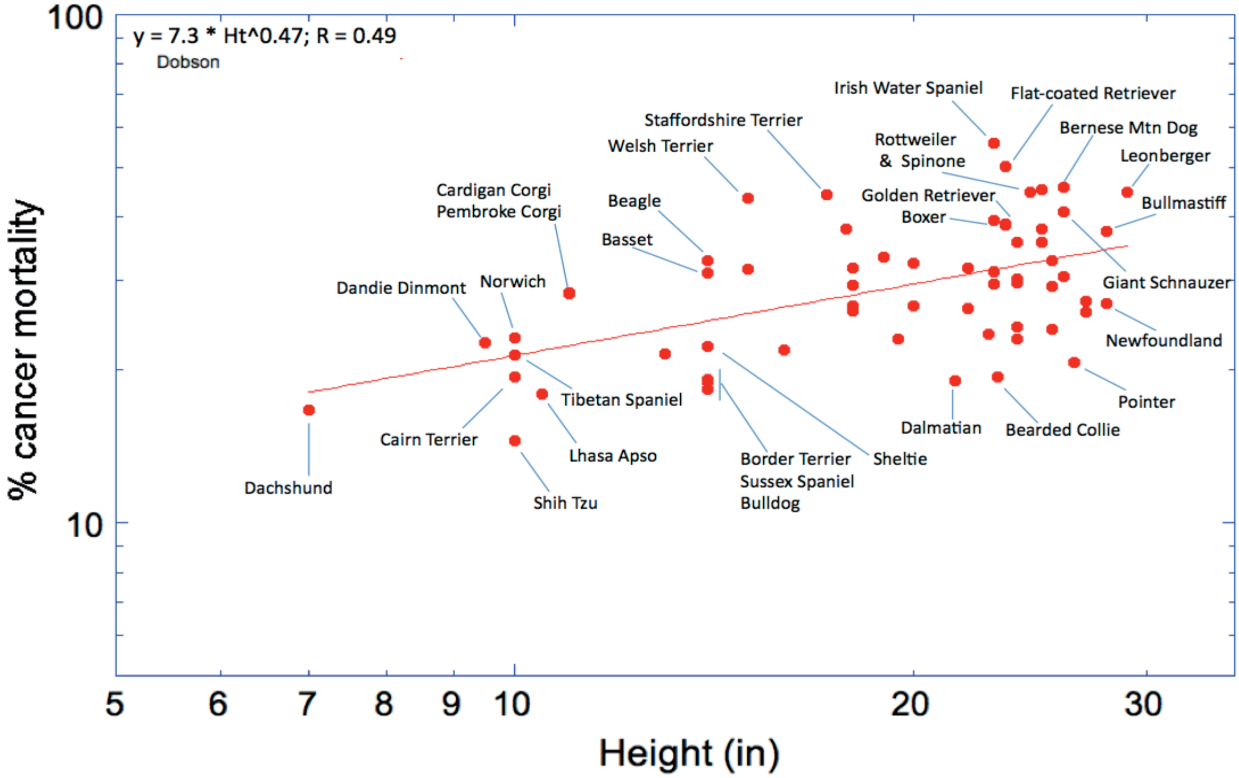

Figure S11 Relationship between height and cancer mortality in dogs.

Data plotted from Dobson, 2013,[3] by Carol Beuchat, PhD
https://www.instituteofcaninebiology.org/lifespan.html

The evolutionary biology of gibbons: F = MA

The various species of gibbons range from 5 to 11 kg in body weight, on average a little larger than the 7 kg for an adult Rhesus macaque. But compared to the 18-year lifespan of the macaque in the wild, gibbons in the wild live twice as long.[4] Thus, despite having many of the principal components of the kill switch in place (albeit different mutations inactivating UOX compared to the *hominidae*), gibbons favored increased lifespan rather than dramatic increases in body size. Why? The simplest explanation is that their method of locomotion, brachiation, selected against such increases in size. Thus, brachiation is a form of locomotion in which gibbons leap from branch to branch, high in the canopy, at speeds of up to 56 km per hour, bridging gaps in the canopy by leaps of as much as 15 meters (50 feet), using gravity as a tool to accelerate their motion. Gibbons do make miscalculations during such locomotion, and their relatively small body size prevents the force of impacting the earth during such accidents from reaching lethal levels. Maintaining the smallest body size among the Hominidae thus meant that gibbons could increase their lifespan in their species-specific solution of the *lex naturalis*. Gibbons also have a very different diet from orangutans and gorillas, eating primarily fruit, and insects. Insects have been shown to concentrate PAH from the environment to the point that levels of PAH contaminating honey have recently been proposed as an accurate measure of PAH contamination of local environments. [5,6] Inclusion of insects as a major feature of the gibbon diet may have increased E in these species, acting as an additional pressure against increased S.

Exception to the rule: Gigantism in Strepsirrhine primates

Archaeoindris fontoynontii was a species of giant lemur that grew to approximately the weight of extant gorillas. It became extinct soon after humans entered Madagascar, about 350 BCE. As we have noted, Strepsirrhine primates have much lower levels of circulating DHEAS than Haplorrhines (Supplemental Figure S1), and we have made the case that such lower DHEAS levels, as well as the absence of the other elements of the kill switch observed in the hominoids, disabled the opportunity of Strepsirrhines to use dramatic increases in body size to exploit new niches. The presence of a large Strepsirrhine primates like *Archaeoindris fontoynontii* in the fossil record therefore requires explanation.

Few fossil remains of this species have been found, indicating that it was a rare form (possibly the only monotypic lemur taxa) occurring during the remarkably diverse lemur speciation that occurred on Madagascar. From the rarity of its fossil remains, and the fact that its dentition shows it to have been a leaf eater, we propose that *Archaeoindris fontoynontii* may have exploited a very specific niche; that is, it may have been very specialized in its food source. Madagascar is well known to have evolved independently from the African mainland for millennia, forming many species of endemic flora and fauna. Some of the flora endemic to Madagascar have been demonstrated to have anti-neoplastic properties, the best known examples of which are the bis-indole alkyloids vincristine and vinblastine, isolated from the periwinkle plant endemic to Madagascar.[7] Many additional potentially bioactive compounds have recently been isolated from plant species found only on Madagascar, and these are only just beginning to be analyzed.[8] A large literature has developed exploring the potential of plant-derived bioactive substances as cancer preventatives.[9,10,11] The possibility therefore exists that *Archaeoindris fontoynontii* may

have attained its great size by the specialized consumption of a plant species that reduced E to a very small value. The *lex naturalis* also suggests that *Archaeoindris fontoynontii* may have had a relatively short lifespan for a creature of its size, a possibility that increases in probability if its specific food source was plentiful, and relatively unexploited by other species. The great size attained by this one-off species may thus derive explanation by a combination of significant reduction in E, and a very restricted capacity for increasing Li (Figure S12)— giving it an abnormally large S/Li ratio as compared, for example, to gorillas and orangutans.

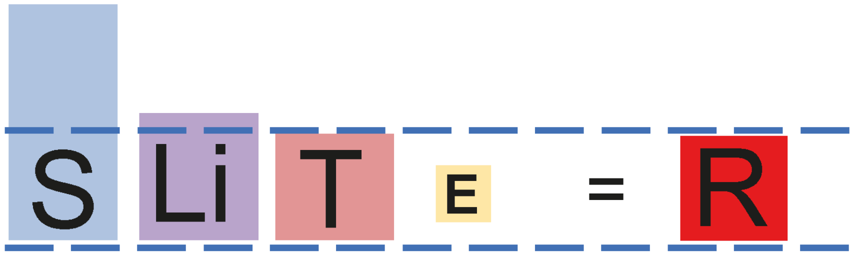

Figure S 12. The *lex naturalis* equation predicted for *Archaeoindris fontoynontii.* It suggests dietary dependence upon a plant species with bioactive properties, and significant limitation on increasing lifespan, Li.

This example demonstrates the potential of the *lex naturalis* equation to illuminate dimensions of fossilized remains unatainable by other means.

References for Supplementary Materials follow

[1] In addition to their increased overall lifetime cancer risk for all types of cancer, tall dogs such as the Irish Wolfhound have a *particularly* high risk for certain kinds of cancer, such as osteosarcoma (Sakthikumar S. et al., 2018 SETD2 is recurrently mutated in whole exome sequenced in canine osteosarcoma. Cancer Research 78(13):3421-3431. doi: 10.1158/0008-5472.CAN-17-3558; Davis BW and Ostrander EA 2014 Domestic dogs and cancer research: A breed-based genomics approach. ILAR J 55(1):59-68. doi:10.1093/ilar/ilu017 and references therein). In both dogs and humans, osteosarcoma generally occurs in the growth plates of long bones. But there are many differences between canine and human osteosarcoma, as well. In humans, osteosarcoma occurs primarily in children, whereas in dogs, the median age of developing osteosarcoma is about seven years, which corresponds to the adult phase of the canine life cycle. Human osteosarcoma occurs more frequently in males, whereas there is no sex preference in canine osteosarcoma. Another difference between human and canine osteosarcoma is that this particular tumor type is 30-50 times more frequent in dogs than it is in humans (Anfinsen KP et al., Breed-specific incidence rates of canine primary bone tumors— A population-based survey of dogs in Norway. Can J Vet Res 75(3):209-215). As exemplified by the Irish Wolfhound, large and giant breeds of dogs account for 90% of all cases of canine osteosarcoma (Dobson JM 2013 Breed predispositions to cancer in pedigree dogs. ISRN Vet Sci 2013:941275. doi: 10.1155/2013/941275). Clearly, the genes that have undergone artificial selection to increase size in dogs has left them with an increased lifetime risk of cancer in general, and osteosarcoma in particular. Fibroblast Growth Factor 1 (FGF1) and its receptor (FGF1R) are associated with many cancers (Ishiwata T 2018 Role of fibroblast growth factor receptor-2 splicing in normal and cancer cells. Front Biosci (Landmark Ed). 2018 Jan 1;23:626-639), and serum FGF1 has been

shown to increase with increasing body size in dogs (Jimenez AG 2016 Physiological underpinnings in life-history trade-offs in man's most popular selection experiment: the dog. J Comp Physiol B. 2016 Oct;186(7):813-27. doi: 10.1007/s00360-016-1002-4. Epub 2016 May 24), offering an explanation as to why large breeds of dogs have an increased lifetime risk of osteosarcoma. But there may also be a temporal aspect to the increased risk of osteosarcoma in the largest dog breeds. Thus, when we forced increases in height in our largest dogs, a corresponding increase in the speed at which they achieved their adult size also occurred in many, but not all of them. It now appears that it is the largest dogs with the shortest developmental period to reach adult height that seem to be at highest risk for osteosarcoma. It thus may be the rapidity of growth in the long bones of big dogs that underlies such high risk(Kent MS, Burton JH, Dank G, Bannasch DL, Rebhun RB. 2018 Association of cancer-related mortality, age and gonadectomy in golden retriever dogs at a veterinary academic center (1989-2016). PLoS One 13(2):e0192578. doi: 10.1371/journal.pone.0192578).

[2] Jones Paul, Chase Kevin, Martin Alan, Davern Pluis, Ostrander Elaine A. Lark Karl G. 2008 Single-Nucleotide-Polymorphism-based association mapping of dog stereotypes. Genetics 179(2):1033-1044. doi: 10.1534/genetics.108.087866 https://www.ncbi.nlm.nih.gov/pmc/articles/PMC2429857/

[3] Dobson Jane M 2013 Breed-dispositions to cancer in pedigree dogs. ISRN Veterinary Science 2013Article ID 941275. http://dx.doi.org/10.1155/2013/941275

[4] Wisconsin Primate Center: http://pin.primate.wisc.edu/factsheets/entry/lar_gibbon/behav

[5] Gavrilović A, Ilijin L, Mrdaković M, Vlahović M, Mrkonja A, Matić D, Perić-Mataruga V 2017 Effects of benzo[a]pyrene dietary intake to antioxidative enzymes of Lymantria dispar (Lepidoptera: Lymantriidae) larvae from unpolluted and polluted forests. Chemosphere.

2017 Jul;179:10-19. doi: 10.1016/j.chemosphere.2017.03.083.
https://www.ncbi.nlm.nih.gov/pubmed/28355530

[6] Al-Alam J, Fajloun Z, Chbani A, Millet M 2019 Determination of 16 PAHs and 22 PCBs in honey samples originated from different regions of Lebanon and used as environmental sentinel. J Environ Sci Health A Tox Hazard Subs Environ Eng 54(1)9-15. doi: 10.1080/10934529.2018.1500782.
https://www.ncbi.nlm.nih.gov/pubmed/?term=Determination+of+16+PAHs+and+22+PCBs+in+honey+samples+originated+from+different+regions+of+Lebanon+and+used+as+environmental+sentinel

[7] Hou Y, Harinantenaina L 2010 New and bioactive natural products isolated from Madagascar plants and marine organisms. Curr Med Chem. 2010;17(12):1191-219.
https://www.ncbi.nlm.nih.gov/pubmed/20158472

[8] Dai Y, Liu Y, Harinantenaina Rakotondraibe L 2018 Novel bioactive natural products isolated from Madagascar plants and marine organisms (2009-2017). Chemical and Pharmaceutical Bulletin 66 (5):469-482 https://doi.org/10.1248/cpb.c17-00395

[9] Dybkowska E, Sadowska A, Świderski F, Rakowska R, Wysocka K. 2018 The occurrence of resveratrol in foodstuffs and its potential for supporting cancer prevention and treatment: A review. Rocz Panstw Zakl Hig 69(1):5-14
https://www.ncbi.nlm.nih.gov/pubmed/29517181

[10] Romagnolo DF, Selmin OL 2012 Flavanoids and cancer prevention: A review of the evidence. J Nutr Gerontol Geriatr. 2012;31(3):206-38. doi: 10.1080/21551197.2012.702534. https://www.ncbi.nlm.nih.gov/pubmed/22888839

[11] Ferrari N, Tosetti F, De Flora S, Donatelli F, Sogno I, Noonan DM, Albini A. 2011 Diet-derived phytochemicals: From cancer chemoprevention to cardio-oncological prevention.

Curr Drug Targets. 2011 Dec;12(13):1909-24.

https://www.ncbi.nlm.nih.gov/pubmed/21158708
